# Ferredoxin, in conjunction with NADPH and ferredoxin-NADP reductase, transfers electrons to the IscS/IscU complex to promote iron–sulfur cluster assembly^☆^

**DOI:** 10.1016/j.bbapap.2015.02.002

**Published:** 2015-09

**Authors:** Robert Yan, Salvatore Adinolfi, Annalisa Pastore

**Affiliations:** King's College London, Institute of Psychiatry, Psychology and Neuroscience, Division of Basic and Clinical Neuroscience, London, UK

**Keywords:** Electron transfer, Enzymology, Friedreich's ataxia, Iron–sulfur cluster biogenesis, Metabolic machines

## Abstract

Fe–S cluster biogenesis is an essential pathway coordinated by a network of protein–protein interactions whose functions include desulfurase activity, substrate delivery, electron transfer and product transfer. In an effort to understand the intricacies of the pathway, we have developed an *in vitro* assay to follow the ferredoxin role in electron transfer during Fe–S cluster assembly. Previously, assays have relied upon the non-physiological reducing agents dithionite and dithiothreitol to assess function. We have addressed this shortcoming by using electron transfer between NADPH and ferredoxin-NADP-reductase to reduce ferredoxin. Our results show that this trio of electron transfer partners are sufficient to sustain the reaction in *in vitro* studies, albeit with a rate slower compared with DTT-mediated cluster assembly. We also show that, despite overlapping with the CyaY protein in binding to IscS, Fdx does not interfere with the inhibitory activity of this protein. We suggest explanations for these observations which have important consequences for understanding the mechanism of cluster formation. Cofactor-dependent proteins: evolution, chemical diversity and bio-applications.

## Highlights

• We have coupled ferredoxin (Fdx) function in Fe-S cluster biogenesis to electron transfer between NADPH via Fdx-NADP-reductase.
• Our results show that this trio of electron transfer partners is sufficient to sustain the reaction *in vitro*.
• Despite sharing the same binding site on IscS, Fdx does not interfere with the inhibitory activity of CyaY.
• These observations have important consequences for understanding the mechanism of cluster formation.

## 1 Introduction

Iron–sulfur (Fe–S) clusters are prosthetic groups crucial for electron transport in respiration and photosynthesis as well as for providing redox potential to numerous enzymes [1]. Cluster assembly is achieved by tightly regulated machines conserved in most organisms. Much of what is known about cluster biogenesis comes from the study of the *Escherichia coli* ISC operon [2], which has close orthologues in primates. Cluster assembly is initiated by IscS, a pyridoxal phosphate (PLP) dependent desulfurase enzyme that converts cysteine to alanine and sulfur. The sulfur generated by this reaction is captured as a persulfide intermediate on an active site cysteine in IscS [3–6]. In Fe–S cluster assembly, the sulfur is transferred to the scaffold protein IscU. The addition of a second sulfur and two ferrous ions leads to the formation of a [2Fe–2S] cluster coordinated by three cysteines of IscU and the active site cysteine of IscS [7].

The speed of cluster formation seems to be under the control of CyaY, the bacterial orthologue of human frataxin [8]. The significance of frataxin function is central to understanding Friedreich's ataxia, a neurodegenerative disorder caused by a gradual depletion of mature frataxin in sufferers who carry mutations in both frataxin genes [9]. Depletion of frataxin causes an imbalance in iron homeostasis, leading to the formation of insoluble Fe^3 +^ deposits which are toxic to the cell and cause oxidative damage. This imbalance in iron homeostasis is likely linked to the frataxin role in regulating the speed of Fe–S cluster assembly.

Current methods for studying the kinetics of Fe–S cluster assembly *in vitro* rely on chemical reducing agents which are used to cleave the persulfide intermediate, thereby catalyzing its release and transfer to the assembling Fe–S cluster. It has been well documented that different reducing agents (dithiothreitol (DTT), β-mercaptoethanol (β-ME), tris(2-carboxyethyl)phosphine (TCEP)), promote cluster formation through different mechanisms and consequently give rise to different kinetics [4]. Since these reducing agents are not physiological, the choice of which to use *in vitro* is arbitrary. Without any scientifically rigorous justification supporting the choice of a specific reducing agent, and given their different effects on the kinetics of Fe–S cluster formation, the significance of findings from these *in vitro* assays regarding the kinetics of Fe–S cluster assembly *in vivo* is strongly questionable.

To overcome this limitation, a better approach would be to use biologically relevant material in place of these chemical reducing agents. It has long been hypothesized that ferredoxin (Fdx) is the physiological reducing agent *in vivo* [1,10]. Fdx, which is also present in the *E. coli* ISC operon, when genetically knocked out, causes disruption in Fe–S cluster assembly [11–14]. Fdx contains itself a [2Fe–2S] cluster (holo-Fdx), that can be in an oxidized ([2Fe–2S]^2 +^) or reduced ([2Fe–2S]^1 +^) state. Switch between the two states can be facilitated by the action of ferredoxin-NADP-reductase (FNR) and NADP/NADPH [15]. It is therefore conceivable that transfer of electrons from reduced Fdx to IscS provides the reductive potential *in vivo* in place of chemical reducing agents although no final proof is currently available.

In recent years, a number of advances have been made in understanding the role of Fdx in Fe–S cluster assembly. Biophysical techniques have established the structure of the IscS/Fdx complex as well as determined their binding affinity [16]. Modeling of the complex using experimental restraints indicates that Fdx binding to IscS positions its [2Fe–2S] in proximity to the active site cysteine of IscS, which could facilitate electron transfer to the assembling Fe–S cluster. Fdx binding in the context of other IscS binding partners, namely IscU and bacterial frataxin, has also been explored. It was shown that Fdx and CyaY interact with IscS in a mutually exclusive manner [16,17]. Conversely, the ternary complexes IscS/IscU/CyaY and IscS/IscU/Fdx can freely form [16,18]. Structural analysis showed that CyaY and Fdx binding surfaces on IscS overlap considerably, but are both distinct from the binding surface for IscU [16].

From a functional perspective, transfer of electrons from Fdx has been shown, albeit indirectly, and in a dithionite (DT) dependent manner [17]. In these experiments it was shown that DT-reduced Fdx converts to oxidized Fdx in the presence of IscS and cysteine. Whilst encouraging, these results however failed to show that Fdx directly promotes Fe–S cluster formation and still rely on the use of DT, a non-physiological chemical reducing agent.

To address these issues, we have set out to prove that Fdx transfers electrons to promote Fe–S cluster formation. The goals of this work are thus threefold: 1) to establish that Fdx functions in conjunction with FNR and NADPH to transfer electrons to promote Fe–S cluster assembly in the absence of non-physiological chemical reducing agents, 2) to develop an *in vitro* assay to study the kinetics of Fe–S cluster assembly using only biologically relevant material to gain better insights into the *in vivo* kinetics of Fe–S cluster assembly and 3) to reassess the effect of CyaY on the kinetics of Fe–S cluster assembly in the new, physiologically relevant assay.

## 2 Materials and methods

### 2.1 Cloning

*E. coli* FNR (UniProt accession number P28861) was procured with *E. coli* codon optimization from GENEART. This was subsequently cloned into pETM-11 using 5′ NcoI and 3′ NotI restriction digest to give a final construct with an N-terminal 6xHis tag and tobacco etch virus (TEV) protease recognition sequence under the control of a T7 promoter.

### 2.2 Sample production

*E. coli* IscS, IscU, Fdx, CyaY and FNR were then expressed and purified as described previously [16,18,19]. The presence of PLP, 2Fe–2S and FAD cofactors associated with IscS, holo-Fdx and FNR respectively was confirmed by UV–vis spectral measurement. In the case of holo-Fdx, A_458_/A_280_ with a cutoff of at least 0.45 was used to confirm high purity of holo-Fdx. After purification, the samples were degassed by purging with argon, aliquoted, flash frozen and stored at − 20 °C.

### 2.3 Fe–S reconstitution assays

Fe–S reconstitution assays were conducted as described previously [20]. Reactions were set up under nitrogen in an anaerobic chamber (Belle Technology) in 1 ml sealable QS quartz cuvettes. Reaction mixtures contained 50 μM IscU, 25 μM Fe^2 +^, and 1 μM IscS and where indicated in the results, 3 mM DTT, 1 μM holo-Fdx, 1 μM FNR, 100 μM NADPH (SIGMA) and/or 1–5 μM CyaY in a final volume of 800 μl in 20 mM HCl–Tris, 150 mM NaCl at pH 8. Reactions were started by the addition of 250 μM cysteine. Reactions were followed by either recording UV–vis scans (800–250 nm) with a Varian spectrophotometer at regular time intervals or by following the CD at 435 nm (CD_435_) with time on a Jasco CD spectrometer. Reactions were carried out at 25 °C and all buffers were degassed by purging thoroughly with argon. Data were fitted to an equation for exponential rise to maximum given by *Abs = C + A(1 − e^− kt^)*, where *Abs* is the absorbance at 456 nm (A_456_) or CD_435_, *C* is the offset from 0, *A* is the amplitude of the curve, *k* is the rate constant and *t* is the time.

## 3 Results

### 3.1 The presence of Fdx/FNR/NADPH is necessary/sufficient to assist cluster assembly

**Fig. 1:**
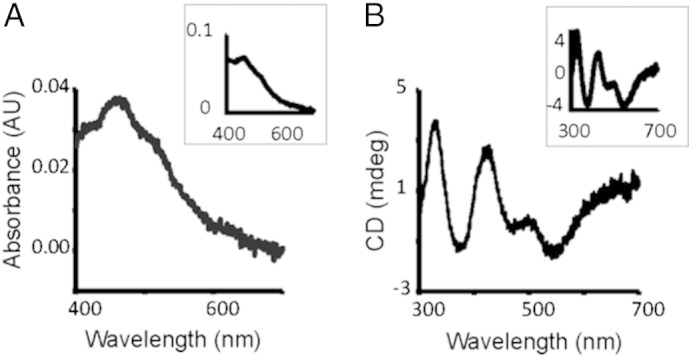
Comparison between Fdx/FNR/NADPH- and DTT-mediated cluster assembly on IscU. UV–vis spectra (A) and CD spectra (B) were recorded after 30 min following the addition of 250 μM L-cysteine to reaction mixtures containing 50 μM IscU, 1 μM IscS, 25 μM Fe^2 +^, 1 μM holo-Fdx, 1 μM FNR and 100 μM NADPH. Inset in (A) and (B) shows the spectra when 3 mM DTT was used instead of Fdx/FNR/NADPH.

**Fig. 2:**
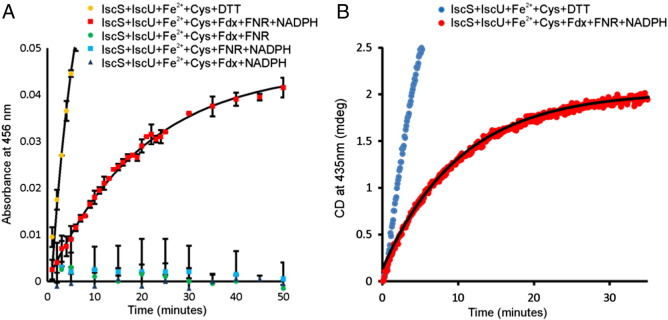
Kinetics of Fe–S reconstitution monitored by UV–vis absorption and circular dichroism. Reactions were started by adding 250 μM L-cysteine to reaction mixtures containing 50 μM IscU, 1 μM IscS, 25 μM Fe^2 +^, and 3 mM DTT or 1 μM holo-Fdx, 1 μM FNR and/or 100 μM NADPH, as indicated in the legends, in sealable QS quartz cuvettes under anaerobic conditions. (A) Following the addition of cysteine, UV scans were recorded at regular time points and the absorption at 456 nm was plotted as a function of time. (B) Following the addition of cysteine, the CD signal at 435 nm was monitored as a function of time. Solid lines in both (A) and (B) denote fits of the data to a function for an exponential rise to maximum.

To follow the kinetics, we recorded UV–vis absorption scans at regular time intervals. The spectral changes as the reaction progressed were characteristic of the formation of [2Fe–2S] clusters on IscU as observed when DTT is used, with an absorption peak at 456 nm (Fig. 1A). Plotting the change in A_456_ with time gave a progress curve that could be fitted to an exponential rise (Fig. 2A). Omission of either holo-Fdx, FNR or NADPH from the reaction mixture completely abolished the formation of Fe–S cluster. This demonstrates that electrons are transferred from NADPH via FNR to Fdx, thus allowing Fdx to deliver electrons to Fe–S cluster assembly in place of DTT.

Since non-Fe–S cluster Fe-species absorb at wavelengths overlapping those for [2Fe–2S] clusters, we also used CD to follow cluster formation and have independent confirmation of our conclusions. The Fdx-mediated reaction gave a CD spectrum characteristic of a [2Fe–2S] cluster, as observed for the DTT-mediated reaction (Fig. 1B). Plotting the change of CD_435_ as a function of time gave a progress curve that could be fit to an exponential rise (Fig. 2B). Taken together, these results show that Fdx, in conjunction with NADPH and FNR, can promote [2Fe–2S] cluster assembly in place of DTT.

### 3.2 Fdx assisted reaction is slower than the DTT assisted one

We next compared the kinetics of Fdx-mediated cluster assembly with DTT-mediated cluster assembly. Both reactions could be fit to an exponential function (Fig. 2A and B). However it was found that the rate of reaction of Fdx-mediated assembly (*k* = 0.05 s^− 1^) was ca. 3 times slower than DTT-mediated assembly (*k* = 0.16 s^− 1^). We tested the effects of increasing the amounts of Fdx, FNR to 5 μM and/or NADPH to 500 μM and 1000 μM but this had no significant effect on the kinetics of the reaction (data not shown). This result suggests that Fe–S cluster assembly *in vivo* occurs more slowly than typically reported for DTT-mediated cluster assembly *in vitro*.

### 3.3 Assessing the role of CyaY in the reaction

**Fig. 3:**
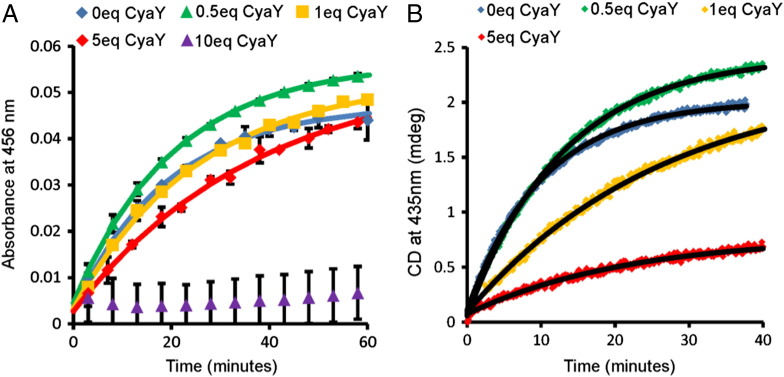
The effects of CyaY on the kinetics of Fdx/FNR/NADPH-mediated [2Fe–2S] cluster formation. Reactions were carried out as described in Fig. 2 and followed by UV absorption at 456 nm (A) and CD at 435 nm (B) with 0, 0.5, 1, 5 or 10 μM CyaY (0–10 eq CyaY) as indicated in the figure legends. Solid lines in both (A) and (B) denote fits of the data to a function for an exponential rise to maximum.

We then decided to reassess the effect of CyaY in the new assay conditions given that the binding sites of this protein and Fdx overlap [16,18,19]. Previously it was shown that CyaY inhibits cluster formation in DTT-mediated Fe–S cluster assembly [20]. When we repeated the experiment using Fdx/FNR/NADPH in place of DTT, we found that the kinetics of cluster formation was further slowed down by the presence of CyaY (Fig. 3A and B). The effect was less pronounced when observing the A_456_ signal compared with the CD_435_ signal. We can exclude that the differences between the results obtained by the two techniques are due to an increased formation of [4Fe4S] which is almost silent in CD but not in UV–vis over [2Fe2S] because the absorption spectra remain those typical of [2Fe2S] clusters (data not shown). The difference is thus likely because non-Fe–S cluster Fe-species also absorb in this region, which masks the effect of CyaY inhibition, highlighting the importance of using CD to monitor cluster formation. It is nevertheless clear that CyaY inhibits [2Fe–2S] cluster formation in Fdx-mediated assembly.

## 4 Discussion

Understanding how Fe–S clusters are produced in the cell is an important topic in biology given the essential role that these prosthetic groups have for all living cells. An important step in this study is to determine the individual role of each of the components of the machines responsible for cluster biogenesis. After the desulfurase and the scaffold protein on which the cluster is assembled, Fdx is probably the most important player given its conservation and its potential importance as an electron donor. Here, we have shown for the first time that the desulfurase reaction can proceed in the absence of other reducing agents strongly suggesting that the function of Fdx (together with FNR and NADPH) is to provide electrons for the reaction. It could, of course, be argued that the reaction requires cysteine and that this could itself function as a reducing agent. If this occurred, it would anyway be inefficient as demonstrated by our controls. Fdx could also play a role in keeping IscU in a reduced state but this cannot be its only function because it would hardly explain why Fdx binds IscS and why this occurs at a site directly adjacent to the active site of the enzyme [16].

Given that Fdx and CyaY occupy overlapping binding site on IscS, we addressed the question as to how they are able to carry out their separate functions in Fe–S cluster assembly. We noticed a strong disagreement between the effects observed by CD and absorbance spectroscopy in the presence of increasing concentrations of CyaY. A likely explanation for this discrepancy is that absorption detects different iron complexes at wavelengths very close to those typical of Fe–S clusters. Frataxin is itself able to coordinate iron and absorbs in this range as previously demonstrated [18,19]. When CyaY binds to IscS, it must release iron since the presence of this cation on its surface would be incompatible with complex formation due to the electrostatic complementarity of recognition (a positively charged surface of IscS binds the conserved negatively charged surface of CyaY) [18,19]. This implies that iron binding on CyaY could mask the spectrum of Fe–S cluster formation when the concentration of CyaY is insufficient to compete out Fdx, making the effect of competition between the two proteins less evident [18,19]. Nevertheless, we observe that, at low concentrations of CyaY, the reaction is under the control of Fdx and proceeds with a rate that is similar to that observed in the absence of this protein in agreement with the previously estimated binding constants. The situation is reverted at increasing concentrations of CyaY at which inhibition becomes evident.

**Fig. 4:**
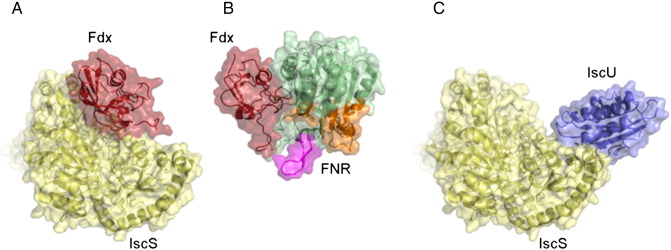
Structures of ISC complexes. (A) HADDOCK structure of the *E. coli* Fdx/IscS complex. (B) Crystal structure of the maize leaf Fdx/FNR complex (pdb: 1GAQ) [21]. The complex is oriented such that Fdx is aligned with the orientation of Fdx in (A). The complex is oriented such that IscS is aligned with the orientation of IscS in (A). The regions of FNR which would potentially clash with IscS and IscU in a hypothetical IscS/IscU/Fdx/IscU ternary complex are circled in brown and blue respectively.

Why do CyaY and Fdx compete for the same site and how could regulation occur? Different scenarios are possible. The two proteins could bind sequentially, displacing each other at different stages of the reaction cycle. Displacement of Fdx from IscS is undoubtedly necessary because of the manner by which Fdx is reduced by NADPH/FNR. The surface of Fdx that binds IscS overlaps with the surface it uses to bind FNR when it accepts an electron (Fig. 4) [21]. FNR would thus be unable to bind Fdx when Fdx is bound to IscS because of steric clashes with both IscS and IscU. For each reaction cycle in Fe–S cluster assembly, Fdx must dissociate from IscS to accept an electron from FNR. In the cycle, CyaY could facilitate the release of Fdx from IscS by competition. Given the co-operativity in binding between IscS, IscU and CyaY [18], CyaY could also have a role in holding the IscS/IscU complex together mid cycle to limit dissociation and release of the unfinished [2Fe–2S] cluster until another reduced Fdx becomes available, thus limiting the release of toxic iron into the cell. An alternatively fascinating possibility is that, since IscS is a dimer, Fdx and CyaY could conceivably occupy one binding site each, thus allowing them to carry out their functions in parallel. The situation *in vivo* becomes even more complicated because IscS is also involved in other metabolic pathways, such as molybdenum cofactor biogenesis and t-RNA thiolation. In these pathways, TusA and ThiI, respectively, bind to the same binding site on IscS as Fdx and CyaY. There are therefore several different proteins competing for IscS indicating the necessity for these interactions to be transient to allow rotation. More work will be needed to clarify this complex and yet fascinating field.

The results described here can certainly be extended to the eukaryotic orthologues given the extent of conservation of the proteins involved. They could also inspire new investigations on how ferredoxins work in other machines.

In summary, our results show that Fdx is responsible for electron transfer in Fe–S cluster biogenesis, the kinetics of Fdx-mediated cluster assembly are slower compared with non-physiological DTT-mediated cluster assembly, and CyaY inhibits cluster assembly in Fdx-mediated cluster assembly in bacteria. Our results lay the foundations for future work to understand the complexity of Fe–S cluster assembly.

## Note added in proof

While this work was submitted, a paper on a related topic was published (Functional reconstitution of mitochondrial Fe/S cluster synthesis of Isu1 reveals the involvement of ferredoxin. Nature Communications, Volume 5). While describing a different perspective of the role of ferredoxin in iron-sulfur cluster biogenesis, this paper is in broad agreement with our conclusions in that electron transfer between eukaryotic ferredoxin, adrenodoxin reductase and NADPH promotes eukaryotic Fe-S cluster biogenesis in vitro in the absence of a chemical reducing agent.

## Conflict of interest

We declare no conflict of interest.
